# A standard set of outcome measures for the comprehensive assessment of oral health and occlusion in individuals with osteogenesis imperfecta

**DOI:** 10.1186/s13023-024-03308-5

**Published:** 2024-08-13

**Authors:** L. Blokland, H. Arponen, A. Ahmad, S. Colijn, H. Gjørup, R. John, M. Li, D. Mekking, S. Parekh, J. M. Retrouvey, T. Stutz Steiger, L. Zhou, K. Andersson

**Affiliations:** 1 Vogellanden, Center of Rehabilitation Medicine and Special Care in Dentistry, Zwolle, The Netherlands; 2 Department of Oral and Maxillofacial Diseases, University of Helsinki, Helsinki, Finland; 3 Helsinki University Hospital Head and Neck Center, Helsinki, Finland; 4 Whittington Health NHS Trust Dental Services, London, UK; 5 Care4BrittleBones Foundation, Wassenaar, The Netherlands; 6 Elkerliek Hospital, Helmond, The Netherlands; 7 Center for Oral Health in Rare Diseases, Department of Dental and Maxillofacial Surgery, Aarhus University Hospital, Aarhus, Denmark; 8 Department of Paediatric Dentistry, University of Bristol, Bristol, UK; 9 Department of Stomatology, The University of Hong Kong-Shenzhen Hospital, Shenzhen, China; 10 Department of Paediatric Dentistry, UCL Eastman Dental Institute, London, UK; 11 Department of Molecular Genetics, Baylor School of Medicine, Houston, TX USA; 12 ProRaris, Vuarrens, Switzerland; 13 Mun-H-Center, National Orofacial Resource Centre for Rare Diseases and Clinic of Pedodontics, Public Dental Service, Region Västra Götaland, Göteborg, Sweden; 14 Department of Pediatric Dentistry, Institute of Odontology, Sahlgrenska Academy, University of Gothenburg, Göteborg, Sweden; 15 Department of Dental Medicine, Division of Orthodontics and Pediatric Dentistry, Karolinska Institutet and Center for Pediatric Oral Health Research, Stockholm, Sweden

**Keywords:** Osteogenesis imperfecta, Dentinogenesis imperfecta, Malocclusion, Outcome measures, Measuring instruments

## Abstract

**Background:**

Osteogenesis imperfecta (OI) is a group of inherited connective tissue disorders of varying severity characterized by bone fragility. The primary objective of this international multidisciplinary collaboration initiative was to reach a consensus for a standardized set of clinician and patient-reported outcome measures, as well as associated measuring instruments for dental care of individuals with OI, based on the aspects considered important by both experts and patients. This project is a subsequent to the Key4OI project initiated by the Care4BrittleBones foundation which aims to develop a standard set of outcome measures covering a large domain of factors affecting quality of life for people with OI. An international team of experts comprising orthodontists, pediatric dentists, oral and maxillofacial surgeons, and prosthetic dentists used a modified Delphi consensus process to select clinician-reported outcome measures (CROMs) and patient-reported outcome measures (PROMs) to evaluate oral health in individuals with OI. Important domains were identified through a literature review and by professional expertise (both CROMs and PROMs). In three focus groups of individuals with OI, important and relevant issues regarding dental health were identified. The input from the focus groups was used as the basis for the final set of outcome measures: the selected issues were attributed to relevant CROMs and, when appropriate, matched with validated questionnaires to establish the final PROMs which represented best the specific oral health-related concerns of individuals with OI.

**Results:**

Consensus was reached on selected CROMs and PROMs for a standard set of outcome measures and measuring instruments of oral health in individuals with OI.

**Conclusions:**

Our project resulted in consensus statements for standardization oral health PROMs and CROMs in individuals with OI. This outcome set can improve the standard of care by incorporating recommendations of professionals involved in dental care of individuals with OI. Further, it can facilitate research and international research co-operation. In addition, the significant contribution of the focus groups highlights the relevance of dental and oral health-related problems of individuals with OI.

**Supplementary Information:**

The online version contains supplementary material available at 10.1186/s13023-024-03308-5.

## Background

Osteogenesis imperfecta (OI) is a genetic connective tissue disorder, with the characteristic symptoms of bone fragility, recurrent fractures, impaired growth, and resulting short stature [1]. Affected individuals may also present with blue sclera, joint laxity, and dentinogenesis imperfecta (DGI) [1]. OI is traditionally classified into four main types according to clinical and radiographic findings, where type I is mild with blue sclerae, type II is pre- or perinatally lethal, type III is the most severe type associated with survival of the perinatal period, and type IV is of moderate severity [2]. Autosomal dominant mutations in the *COL1A1* and *COL1A2* genes are causative in approximately 85% of cases [3–5]. More recent studies have revealed a plethora of recessive and X-linked mutations in genes related to the production and modification of collagen type I, leading to the proposal of a new OI nomenclature comprising five main types [6]. Significant efforts have also been made to adopt a dyadic naming system by systematically associate the phenotypic entity with the gene it arises from [7]. However, the classification by Sillence is still the most frequently used in clinical practice.

Dental and craniofacial aberrations are common findings in individuals with OI [8–11]. The most common oral findings in OI are DGI and malocclusion such as open bite in the anterior or posterior region of the occlusion [8, 9, 11, 12]. Teeth affected by DGI exhibit a characteristic grey-blue to brown discolouration due to dysplastic dentin [13, 14]. The enamel, which shows normal structure and mineral content, is easily fractured due to the underlying dysplastic dentin, which is prone to attrition [9]. The deciduous dentition is often more severely affected than the permanent dentition [9]. Radiographically the teeth exhibit a deviating morphology, pathognomonic for the condition, with bulbous crowns, a marked cervical constriction, pulpal obliteration, and short roots [11, 13]. DGI is classified into two subgroups based on genetic findings; DGI type I, syndromic form associated with osteogenesis imperfecta and DGI type II, non-syndromic form [14–18]. In individuals with OI, depending on the type, prevalence of DGI is estimated to be between 8 and 100% [8, 12]. More severely affected children often have a more severe dental phenotype [8, 9, 12]. Additionally, many individuals with OI present with other dental aberrations. Among these are dental agenesis, apically extended pulp chambers (taurodontism – in individuals without DGI), retained permanent second molars and abnormal craniofacial development with vertical underdevelopment of dentoalveolar structures [9, 19–23]. The oral manifestations in OI may have a significant impact on oral health-related quality of life [24–27].

The phenotypes seen in OI exhibit an extensive heterogeneity. The plethora of findings, including oral and craniofacial manifestations, can also differ within the same type of OI.

From birth to adult life, the children and adolescents grow and develop both physically and mentally. All ages are associated with the need for special considerations when it comes to securing oral health and monitoring craniofacial development. A solid base of knowledge is mandatory for diagnosis and treatment of traits. Defining a set of reliable and valid outcomes for individuals with OI that would cover all essential oral and craniofacial aspects and be applicable worldwide is challenging. Such an outcome set could potentially improve the standard of care on both an individual and a population level. Standardization of outcome measures is of utmost importance to enable aggregation of data from different studies, to compare data from different data sets, to allow evidence synthesis, and most importantly, be relevant to the individuals affected by the disorder [28]. Individuals with rare diseases are at increased risk of unmet clinical needs due to limited access to information and clinical care [29, 30]. To meet this challenge, the Care4BrittleBones foundation initiated the Key4OI Plus, a project to develop a minimum standard set of outcomes and associated measures for the comprehensive appraisal of OI that would reflect the complexity of OI care and focusses on the issues that are considered most important by individuals with OI [31].

The primary aim of this initiative was to reach an interdisciplinary, international consensus for a standard set of outcomes and measuring instruments for oral healthcare in OI, based on the aspects considered important by both the dental profession and individuals with OI. This standard set is to be comprehensive enough to cover the full range of dental care in OI, as well as practical enough for valid implementation. This approach would permit oral healthcare teams to measure and monitor their performance in a consistent way. Furthermore, this would support longitudinal and cross-sectional comparisons of outcomes between centers that serve OI populations in different countries and cultural contexts.

## Methods

### Participants of the project team

The Care for Brittle Bones Foundation reached out to all main OI patient organizations, including OIFE and OIF to identify any national experts for oral health/dentistry in their respective countries. In addition, the identified experts reviewed literature to ensure all available expertise was invited to join the project team. The project team was composed of thirteen individuals from eight different countries, both patient experts and dental care providers with different specialties in dentistry.

The project was led by a project coordinator (n = 1) from the Care4BrittleBones Foundation. Clinical disciplines represented included pediatric dentistry (n = 5), orthodontics (n = 3), oral and maxillofacial surgery (n = 1) and prosthodontics (n = 1). Furthermore, patient representatives with a professional background in healthcare (n = 2) were represented. Of the professional experts, five were involved in dental care for children and five experts involved in dental care for both children and adults.

### Initiation of the project

The project was initiated and coordinated by the Care4BrittleBones Foundation. The project used the same approach and principles as for the Key4OI Outcome set [31]. For the original Key4OI work an ethical review was conducted and confirmed that no ethical review was required for the development of outcome measures under this project.

The project commenced with a meeting explaining the purpose and voluntary nature of the project participation. A total of thirteen (monthly and later biweekly) virtual consensus meetings were facilitated by the project coordinator, in the period November 2020–January 2022. All meetings were summarized and recorded, in order to address occasional absences of individual members.

### Literature review

In the first phase, the project team collected possible outcome measures related to oral health, dental condition, and occlusion in people with osteogenesis imperfecta, based on available literature and clinical experience. The expert team conducted a non-systematic in-depth literature review of relevant publications in OI and oral health, based on the expertise of each specialist. Variables present in the literature were merged with the extensive collective clinical experience from the expert group. Literature inclusion criteria were original research articles and publications in peer-reviewed journals. Exclusion criteria were articles not available in English or the inability to obtain the full-text article. This resulted in a list of possible clinician-reported outcome measures (CROMs) and expected relevant CROMs. The results were shared in consensus meetings and converted into a comprehensive list of aspects of both CROMs and patient-reported outcome measures (PROMs). Based on the literature, relevant measuring instruments attributing these outcome measures were collected.

### Focus groups

The patient representatives and project coordinator recruited individuals with OI to participate in focus groups. They worked together with various patient organizations to inform the patient community about the upcoming project, and they shared the information on social media. The group consisted of eight male and twelve female patients. The type of OI was distributed as follows: Type I: 6, Type III: 8 and Type IV: 6. To maintain anonymity, complete health records of the focus group participants were not documented. Not all patients consented to record their age. All were above 18 years. The youngest patient was 18 and the oldest 58. The patients were from 11 different countries: two from the US, one from Asia and the others from European origin. The professional experts of the project team were not involved in recruiting the focus groups and did not participate in focus group sessions, to ensure participant anonymity, voluntariness, and unbiasedness.

The project coordinator organized three focus group meetings on 24th of February, 3rd and 9th of March 2021. A total number of 20 adults with OI attended and discussed in the group, supported by a moderator and an innovative IT tool called Mural^©^ (see www.mural.com). It is a well-established and proven platform to engage virtual audiences. The meetings were provided by written comments, a summary based on the written comments composed by the two patient experts of the project team, and recordings of the meeting.

During the sessions, 29 themes mentioned by the participants within the sessions, were discussed. Key themes were defined as themes mentioned in all sessions and they were marked as “top priority”. Themes that strongly resonated in two of the three sessions were marked as “priority”.

The key themes mentioned by the focus groups were explained by the patient experts and discussed in project team meetings. The key themes were compared and attributed to the selected CROMs. The items on the CROMs list were discussed, for: relevance of the outcome measure and applicability (time, languages and costs) and validation (also in different languages) of possible measuring instruments. The CROMs mentioned by the focus groups were considered more relevant, than the CROMs mentioned by the experts only.

A different approach to PROMs was adopted. PROMs measuring instruments consisted of mainly questionnaires. All items of the different questionnaires were split into different items/questions. The key themes of the focus groups were matched with the items of the questionnaires, in order to identify the measuring instruments (i.e. questionnaires) covering most and most relevant (high priority) items.

### Consensus: Delphi rounds

A modified Delphi technique was used to develop a consensus on a minimal standard outcome set of CROMs and PROMs. The Delphi technique is an iterative multi-stage process to actively transform opinion into group consensus [32]. In a Delphi study, anonymous responses of the project team members are aggregated and shared with the group after each round, ultimately leading to a group consensus. The consensus should be based on the data derived from professional experts and individuals with OI themselves.

In a digital platform, commonly used for scientific surveys, experts anonymously rated the proposed outcome measures and measuring instruments for inclusion in the set, on a 9-point scale. A minimum of 80% with a score of seven, eight or nine was required for the final confirmation of each individual component comprising the measuring instrument. A score of one, two or three in 80% of responses lead to a rejection. Mid-range scores of four, five or six were regarded as “non-conclusive”. The anonymous “non-conclusive” responses were re-discussed in the next consensus meeting and tabled in the next Delphi round. A participation of 80% of experts was required in the Delphi rounds [32, 33].

## Results

### Literature review and expert opinion/clinical experience

In the first phase of the project, relevant dental items were identified by the project team. The literature review and expert opinion resulted in 35 articles that were reviewed and discussed by the project team. Table 1 outlines the relevant oral health-related aspect and measuring instruments addressing them identified by the professional experts of the project team.

**Table 1:** List of possible outcome measures, dental items in osteogenesis imperfecta

*Clinician-related outcome measures (CROMS)*
General oral health Hard tissues and oral health
Periodontal health
Tooth wear
Plaque
Osteogenesis imperfecta related oral health
Dentinogenesis imperfecta
Expressivity—Mild/Moderate/Severe
Clinical indicators
Radiographic indicators
Histological assessment
Other dental anomalies Shell teeth
Tooth agenesis
Taurodontism
Pulpal stones
Ectopic molars (impaction/retention), eruption first molars
MIH
Other anomalies
Malocclusion—orthodontics Sagittal incisal relationship
Vertical incisal relationship
Sagittal molar occlusion
Vertical molar occlusion
Transversal molar occlusion
Occlusion and aesthetics
Orthodontic treatment priority
Osteogenesis imperfecta related (medical) contra-indications or barriers with regard to dental treatment
*Patient-reported outcome measures (PROMs)*
Oral pain and dysfunction
Oral health-related quality of life Oral function
Aesthetics
Burden of treatment
Accessibility to dental treatment
Anxiety related to dental treatment

### Focus groups

During the sessions, 29 themes were discussed. A total of 13 themes were addressed as relevant to individuals with OI: 6 themes came out as key themes in all sessions (marked as “top priority”) and 7 additional themes (the other dental items) strongly resonated in 2 of the 3 sessions (Table 2). The themes from the focus groups were reviewed and discussed by the professional experts of the project team and were assessed as being covered by the initially identified dental items. The key themes mentioned by the focus groups were attributed to the relevant CROMs and/or listed as a PROM (Table 3).

**Table 2:** Key themes identified in focus groups across sections

Key themes identified	Priority
Breaking off tooth pieces, chipping, fracturing teeth	Top
Dental implants	Top
Embarrassed because of teeth or mouth	Top
Ignorance of dentists	Top
Experiencing pain (tooth or jaw)	Top
Proportions of jaw and how they "match" (over/under/open/crossbite)	Top
Affordability of dental treatment	
Anxiety in relation to future of jaws/teeth	
Difficulty eating certain type of food	
Earache	
Facings not sticking/coming off or loosening	
Fillings/Crowns	
Root canal issues (infection, disappearance of visible canal)

**Table 3:** Attribution of key themes mentioned by the focus groups to the relevant CROMs and/or listed as a PROM

Key themes identified across sessions	Attribution to CROMs or PROMs
Breaking off tooth pieces, chipping, fracturing teeth	CROMs: OI-related oral health/DGI OI-related oral health or general oral health/tooth wear PROMs: Functional impairments
Dental implants	CROMs: OI-related medical contra-indications or barriers
Embarrassed because of teeth or mouth	CROMs: OI-related oral health/DGI and malocclusion PROMs: Esthetics
Ignorance of dentists	PROMs: separate (new) item
Experiencing pain from teeth or jaws	CROMs: General oral health PROMs: Orofacial pain and dysfunction
Proportions of jaws and how they "match" (over/under/ open/crossbite)	CROMs: Malocclusion PROMs: Esthetics Functional impairment?
Affordability of dental treatment	PROMs: separate (new) item
Anxiety in relation to future of jaws / teeth	PROMs: anxiety Discussion: anxiety for dental treatment or insecurity about situation/possibilities/barriers
Difficulty eating certain type of food	CROMs: General oral health (missing teeth, type of prosthetic appliance, periodontal condition) Malocclusion Breaking/chipping > OI-related oral health/DGI PROMs: Orofacial pain and dysfunction
Earache	CROMs and PROMs Discussion: orofacial pain and dysfunction possible aetiology
Facings not sticking / coming off or loosening	CROMs: OI-related oral health / DGI PROMs: Functional impairment
Fillings/Crowns	CROMs: OI-related oral health/DGI
Root canal issues (infection, disappearance of visible canal)	CROMs: OI-related oral health/radiographic DGI indicators/pulp obliteration

### Selection of outcome measures and measuring instruments

Based on the input of the professional experts of the project team and the focus groups in the previous phases, outcome measures were formulated, and corresponding measuring instruments were collected (Table 4).

**Table 4:** Selection of outcome measures, with input of the focus groups and possible measuring instruments

Domain		Outcome measure	Measuring instruments
*CROMs*			
General oral health		Hard tissues and oral health	DMFT (Decayed Missing Filled Teeth) [65]
		Periodontal health	BPE (Basic Periodontal Examination) CPITN (Community Periodontal Index of Treatment Needs) Periodontal status [34–36, 66, 67]
		Endodontic health (periapical pathology)	Clinical and/or radiographic assessment
		Tooth wear	BEWE (Basic Erosive Wear Examination) [68] TWI (tooth wear index) [69]
		Plaque	Plaque score [37]
		Number of permanent teeth	Clinical or radiographic assessment
		Number of extracted teeth	Clinical or radiographic assessment
		Number of teeth with direct/indirect restorations	Clinical and/or radiographic assessment
		Number of dental implants	Radiographic assessment
		Number of endodontically treated teeth	Radiographic assessment
		Type of dental prosthesis	Clinical assessment: 0. Implant supported crowns 1. Fixed partial denture, dental supported 2. Fixed partial denture, implant supported 3. Removable partial denture 4. Full denture 5. Other?
OI specific oral health /DGI spectrum	DGI—Expressivity	Expressivity	Clinical and radiographic assessment: 3 types of expressivity: 1. Presence in primary dentition 2. Presence in both 3. Isolated histological DGI only
	DGI—Clinical DGI indicators	1. Pathologic discoloration	Clinical assessment—according to scale or description (blue/grey or yellow/brown) [8, 14, 38]
		2. Attrition	BEWE [68] TWI [69]
		3. Fractures	Clinical assessment
	DGI—Radiographic DGI indicators	1. Bulbous crowns with cervical constriction	Radiographic assessment (Y/N) [8]
		2. Pulpal obliteration	Radiographic assessment (Y/N AND partial (Pulp chamber is not visible and the canal is markedly narrowed but visible) or total (the pulp chamber and canal is hardly or not visible))
		3. Small radices / short roots	Radiographic assessment
	DGI—Histological assessment	Histological DGI	Histological examination [60]
OI specific oral health		Shell teeth	Clinical and radiographic assesment
		Tooth agenesis	TAC (Tooth Agenesis Code) [70] Hypodontia/oligodontia Registration of the number and specification of teeth congenitally absent [22, 71]
		Taurodontism	Radiographic assessment (first molars) (Y/N) [12, 69]
		Pulpal stones	Radiographic assessment (Y/N)
		Permanent molar eruption (impaction or retention)	Relevant if it occurs in association with DGI and/or aberrant craniofacial development/malocclusion (Y/N in association with DGI and/or aberrant craniofacial development/malocclusion) Clinical and radiographic assessment. Categorizing by categories: 1. Developing tooth 2. Normal eruption 3. Mesioangular impaction of maxillary molar 4. Retention of maxillary molar (not erupting, no physical obstacles I eruption pathway) 5. Impaction of mandibular molar (due to ectopic position or an obstacle) 6. Retention of mandibular molar (not erupting, no physical obstacles in eruption pathway) [72, 73]
		Molar‑incisor‑hypomineralisation	EAPD classification [74]
		Other anomalies	Clinical and/or radiographic assessment
Craniofacial / Orthodontics		Sagittal incisal relationship	Clinical assessment (overjet in mm OR maxillary overjet (> 5 mm) Y/N and mandibular overjet (= 0 mm or < 0 mm) (Y/N) [75]
		Vertical incisal relationship	Clinical assessment (assesment of overbite in mm OR anterior open bite (< 0 mm) Y/N and deep bite (> 4 mm) Y/N)
		Sagittal molar occlusion	Clinical assessment (occlusion according to Angle classification)
		Vertical molar occlusion	Clinical assessment (lateral open bite in molar region Y/N)
		Vertical molar occlusion	Clinical assessment (lateral open bite in premolar region Y/N)
		Transversal molar occlusion	Clinical assessment (crossbite Y/N? Crossbite uni- or bilateral)
		Orthodontic diagnosis	Radiographic assessment? (orthopantomography, cephalometry, Cone beam CT)
		Occlusion and aesthetics	ICON (Index of Complexity, Outcome and Need) [76]
		Orthodontic treatment prioriy	IOTN (index on treatment need and indication of why the treatment need existst) [77]
*PROMs*			
Pain and dysfunction		Pain, temporomandibular disorders, orofacial pain and dysfunction	DC/TMD [78, 79] 3 questions TMD screening [48, 49] MFIQ [80]
OHRQoL/ aesthetics		Oral function	COHIP-SF 19 [81] CPQ [46, 47, 82] OHIP-49 [45] OHIP-14 [83] according to PROMS AI (SP) [84]
		Aesthetics	OES (Orofacial Esthetic Scale) [85, 86]
Burden of (dental) treatment			No. of appointments, duration, patient perception
Dental anxiety		Anxiety (related to dental treatment)	CEDAM [87] MCDAS [88, 89] Single question with VAS [90]
Accessibility to dental treatment (input focus groups)			–

In the five Delphi rounds, carried out between September 2021 and January 2022, a 100% consensus was reached on a set of oral health-related outcome measures and measuring instruments. A clinical practice guideline recommendation that was considered not to be part of the set but too important to be neglected, was also made. This recommendation was included based on acceptance for inclusion by 10 out of 12 experts (> 80%). The recommended set of outcome measures and measuring instruments is represented in Table 5.

**Table 5:** Standard set of outcome measures and measuring instruments for the assessment of oral health and occlusion of individuals with Osteogenesis Imperfecta

General oral health	Hard tissues and oral health	Adults: DMFT* every routine examination, at least every 2nd year Children: dmft* at least at age 3, 6, 13 (DMFT when permanent teeth have erupted) *Including: reasons for "filled" as follows: (1) tooth wear and/or chipping and/or fracture (non-cariogenic tooth substance loss) (2) caries (3) unknown aetiology	
	Periodontal health	BPE, from age 7 at every routine examination	
OI-specific oral health	Dentinogenesis imperfecta	Y/N (clinical/radiological), 2 baselines: primary (3–6 years of age latest) and permanent (when all have erupted)	
	Dental anomalies	Recording of anomalies (shell teeth, tooth agenesis, taurodontism, ectopic permanent second molars (impaction/retention), eruption of first molars)	
	Occlusion	Horizontal overjet	Horizontal overjet: measurement (if overjet ≥ 0 mm) and clinical assessment of presence of increased horizontal overjet (overjet > 5 mm) Y/N
		Mandibular overjet	Mandibular overjet: clinical assessment of presence of mandibular overjet (overjet ≤ 0 mm Y/N) and measurement (if mandibular overjet ≤ 0 mm)
		Vertical overbite	Vertical overbite: measurement of vertical overbite (if VOB ≥ 0 mm) and clinical assessment of presence of increased vertical overbite (≥ 5 mm) / deep bite Y/N)
		Anterior open bite	Anterior open bite: clinical assessment (VOB < 0 mm Y/N) (3) and measurement (if VOB < 0 mm)
		Crossbite	Crossbite: clinical assessment of presence of crossbite (Y/N) and notification of crossbite type 1 (Unilateral / bilateral / anterior) and/or crossbite type 2 (Molar/premolar-canine / incisor)
		Posterior open bite	Posterior open bite: clinical assessment of presence of crossbite (Y/N) and notification of posterior open bite type 1 (Unilateral / bilateral) and/or posterior open bite type 2 (Molar/premolar-canine)
		Clinical assessment of molar eruption	Clinical assessment of molar eruption
		Any kind of malocclusion	Any kind of malocclusion should be recorded. Special consideration should be put on presence of the previously mentioned variables as they are more prevalent in individuals with OI
	Tooth wear	Identification of the presence of pathological tooth wear (i.e. non-physiological) by recording " (1) yes, (2) possibly, (3) no/physiological When screening for pathological tooth wear make use of the appropriate methods suitable for the specific situation in an individual patient. The use of indices, photographs, study casts, digital 3Ddatasets, clinical examination and anamnestic information can be part of that	
OI-related (medical) contra-indications or barriers with regard to dental Tx	Recording medical treatment with bisphosphonates If bisphosphonates are used: record start and end date of treatment Prior dental procedures/treatment. Clinical assessment and history taking		
Oral Health Related Quality of Life (OHRqOL)		We recommend measurement of Oral Health Related Quality of Life by CPQ 8–10 at age 8 and CPQ11-14 at age 11 We recommend measurement of Oral Health Related Quality of Life by OHIP-49, starting at age 15, every 5 years	
Temporomandibular Disorder (TMD)		In children and adolescents, we recommend measurement (screening) of TMD-problems by 3Q/TMD annually, starting at age 10 In adults (above 19 years), we recommend measurement (screening) of TMD-problems by 3Q/TMD with an interval of 2 years	
Basilar invagination		If radiographic scans (CBCT or Ceph) are made or already exist, the dentist has a responsibility that any anomalies or pathologies are reviewed, including and specifically concentrating on any indications for BI. It is recommended to refer to a radiologist with relevant competence for this assessment	

## Clinician-related outcome measures (CROMS)

The project team recommended to measure general oral health and OI-specific oral health items.

### General oral health

General oral health is recommended to be measured as the number of decayed, missing and filled teeth (DMFT for permanent dentition and dmft for deciduous teeth) and the periodontal health with the screening tools of Basic Periodontal Examination (BPE) and Community Periodontal Index (CPI) [34–36]. In addition, plaque score was included in the assessment of general oral health [37]. Recommended frequency for recording of DMFT was in conjoint with every routine examination but at least every second year, and dmft in the primary or mixed dentition at least at age 3, 6 and 13 years. In addition, consensus was reached for the need to record reasons for the “filled” as either: (1) Tooth wear and/or chipping and/or fracture (non-cariogenic tooth substance loss), (2) Caries and (3) Unknown aetiology. The reasons behind the DMFT/dmft numbers were considered more important and indicative for specific oral problems, than the absolute number.

Measuring periodontal health by Basic Periodontal Evaluation (BPE) from age 7 at every routine examination, was recommended.

### OI-specific oral health

Measurement of OI-specific oral health was divided into 5 items: DGI, other (dental) anomalies, items related to occlusion, tooth wear, and OI-related medical contra-indications or barriers regarding dental treatment.

### Dentinogenesis imperfecta

The presence (yes/no) of DGI should be recorded, based on clinical and/or radiographic indicators [38]. As the presence of DGI does not change with time, but the expressivity may differ significantly between the primary and permanent dentition, two baseline assessments were recommended: first for the primary dentition between 3 and 6 years of age at latest, and second in the permanent dentition, when all the permanent teeth except for wisdom teeth, are present [12].

### Other dental anomalies

Other dental anomalies or relevant dental findings should be recorded. Special emphasis should be put on presence of tooth agenesis, shell teeth (as an early sign of DGI before obliteration of the pulp), taurodontism, ectopic permanent second molars (impaction/retention) and ectopic eruption of first molars, as these anomalies are more prevalent in individuals with OI compared to the general population [9, 11, 12, 22, 39].

### Occlusion

Malocclusion is a common finding in individuals with OI. With regard to occlusion, measurement of horizontal overjet and vertical overbite is recommended, as well as recording the presence of possible open bite, crossbite, ectopic eruption of teeth and/or other occlusal deviation [40, 41].

### Tooth wear

Consensus was reached on the relevance of the assessment of tooth wear in individuals with OI. Little evidence is available, but tooth wear in combination with DGI with accompanying risk for chipping and fractures and the compromised condition of dentin, was presumed to be an important factor in tooth prognosis. No consensus was reached on the index that should be used. However, recommendation included identification of the presence of pathological (i.e. non-physiological) tooth wear by recording: (1) Yes; (2) Possibly; (3) No/physiological. As no consensus could be reached, it was recommended to make use of the appropriate methods suitable for the specific situation in the individual patient. The use of indices, photographs, study casts, digital 3D datasets, clinical examination and anamnestic information can be part of that.

### OI-related medical contra-indications or barriers with regard to dental treatment

Prior to dental procedures or treatment, clinical assessment and history taking should be performed. Medical contra-indications or barriers should be recorded. Especially medical treatment with bisphosphonates is considered relevant. If bisphosphonates are used, start and end date should be recorded. The reason is the potential effects of bisphosphonates on tooth movement in orthodontic treatment, tooth development and medication-related osteonecrosis of the jaw (MRONJ) [42–44].

## Patient-reported outcome measures (PROMs)

It was recommended to measure oral health-related quality of life by CPQ8-10 at age 8, CPQ11-14 at age 11 and OHIP-49 starting at age 15, every 5 years [45–47].

Consensus was reached on measuring (screening) temporomandibular disorders (TMD), by 3 screening questions (3Q/TMD) [48–50]. Screening was recommended to be performed annually starting age 10, and above 19 years with an interval of 2 years.

The Delphi results on ‘anxiety’ were inconclusive. Discussion on this topic led to no views on this topic and anxiety was not included in the final set.

## Recommendation

The importance of assessment of basilar invagination or impression was acknowledged in several discussion meetings. Basilar invagination is a serious co-morbidity of OI that may cause life-threatening compression of medulla and cervical spine [51–53]. Lateral skull radiographs, Conebeam CT (CBCT), or MR/CT-images used for dental/orthodontic assessment (cephalometrics) can reveal asymptomatic cranial base pathologies [52, 54]. However, no consensus could be reached on inclusion and especially measuring methods of BI in this set of oral health-related outcome measures. In the final Delphi, consensus was reached on the following recommendation: If radiographic scans (CBCT or Cephalometric radiographs) are obtained or already exist in patient records, the dentist has a responsibility that in case any anomalies or pathologies are observed, to refer the patient to a radiologist with relevant competence for assessment of craniocervical pathology.

## Discussion

In this Delphi consensus study, we developed a standard set of outcome measures and measuring instruments on oral health and occlusion in individuals with OI, which can be implemented by healthcare professionals all over the world. The standard set of outcome measures proposed in this study enables the assessment and comparison of relevant dental and oral health problems. In addition, systematic implementation of a standard set of outcome measures by oral healthcare professionals can facilitate future research on dental and oral health problems in people with OI.

At present, several centers providing care for individuals with OI have established consensus guidelines on the use of bisphosphonate therapy, physical rehabilitation and surgical management of fractures [55–57]. This is the first oral health-focused guideline project that incorporates patient-reported outcome measures into clinician-related outcome measures reviewed by a panel of individuals with OI and an international group of experts.

Although not included in the Delphi rounds, it is important to note that an oral hygiene inventory should be conducted for all patients during each visit. A consistent daily oral hygiene routine has a substantial impact on individual’s oral health and affects the other outcome variables. The frequency of routine examination was not included in the standard set. The authors acknowledge that regular follow-up on an individually based frequency is of importance, especially when factors are present that may hamper oral health such as a more severe phenotype.

In people with OI, especially when multiple health issues are present, assessment of the oral health may not obtain highest priority [58]. However, the outcome of the focus groups sessions emphasized the importance of dental and oral problems as well as the impact they have on everyday life, especially when oral functional impairments or esthetic problems are present. Focus group discussions stated that dental problems and oral health-related concerns can be left underestimated and that oral health professionals may appear reluctant to act on them. The absence of universal treatment guidelines, limited scientific evidence and knowledge, and limited clinical experience in a rare disorder like OI may contribute to this reluctance [59].

Previous studies have demonstrated that the dental concerns of children and adolescents with OI affect functional and socio-emotional well-being and thereby oral health-related quality of life [24, 27]. This emphasizes the relevance of adequate attention for oral health and oral health-related well-being, in individuals with OI.

### Strengths and limitations

A strength of this study is that the consensus statements were based on input from both focus groups and oral healthcare providers. The input from the focus groups formed the basis for the set of outcome measures. Moreover, the selection of the specific measuring instrument for assessment of oral health-related quality of life, was derived from the focus groups input. The oral healthcare providers, all have a special interest in OI and covered different areas of specialties of dentistry relevant for full assessment in complex dental/oral conditions. The participation of two patient experts, both professionals in healthcare, was considered very valuable. The geographic representation of the experts can be considered a limitation: the expert team involved professionals from Europe, North America and Asia but none from Africa, Oceania, or South America. Another limitation of the study is that a certain degree of computer skills was required to attend the focus group sessions possibly affecting the group composition. Illiterate people and children were not represented. It is possible that those individuals with OI, who experience oral health-related problems were more prone to participate in the focus groups. This possible sampling bias can be also considered a strength of the study as it could be speculated to result in addressing of the relevant dental problems. However, it might also imply a biased view on the dental problems when people without dental problems or positive experience did not participate.

Two of the priority items mentioned in the focus group sessions, were not selected as an outcome measure by the oral health professionals. Both *ignorance of dentists* and *affordability of dental treatment* were rejected based on discussion in the expert team sessions, due to lack of universal methods to measure them for global comparison. Nevertheless, these items are highly important to people with OI.

To date, no OI specific questionnaire exists for Oral Health. Hence, the assessments included in the standard set are not validated for OI. OI is a very heterogeneous disorder and the prevalence is low. Therefore, to develop and implement a validated OI specific questionnaire for Oral Health in OI would be very challenging. The fact that the disorder is unknown for most oral health professionals emphasizes the importance of our study in raising awareness and understanding of oral health issues associated with OI. This study aimed to do this initially using validated non-OI specific questionnaires, as this this approach is scientifically more sound in the short and mid-term. In the long term, developing an OI-specific validated questionnaire is certainly a topic for future research. DGI may be diagnosed by clinical, radiographic and/or histologic findings. In cases of no obvious clinical or radiographic findings, a histologic examination of an exfoliated or therapeutically extracted tooth may still reveal dentin anomalies associated with DGI [12, 60]. Based on this extensive phenotypic heterogeneity (clinically, radiographically, histologically), the expert group discussion led to the suggestion for a more comprehensive classification of OI-related DGI. This would facilitate diagnosis and increase the basis for well-founded treatment guidelines. However, the opportunity for histological examination differs between countries. Based on this, the expert group considered it as not being part of the minimum standard outcome set. The need for a classification of OI-related DGI was acknowledged but considered beyond the scope of this study.

At present, no OI-specific tooth wear index exists that would take into consideration the presence and effect of DGI. In individuals with OI and DGI, the enamel frequently chips from the affected dentin leading to tooth fractures [61]. There is no gold standard in assessment of tooth wear. Although recommendations exist on identification and treatment of pathological tooth wear, it might not be suitable for use in people with DGI [62, 63].

The recommended frequency for recording of DMFT was in conjoint with every routine examination but at least every second year, and dmft in the primary or mixed dentition at least at age 3, 6, and 13 years. In case of adults without registrations and when the cause of missing teeth is not remembered, the adjusted decayed and filled teeth (ADFT) can be applied, to overcome this problem [59, 64].

In the final set of outcome measures and measuring instruments both measurement of horizontal overjet and mandibular overjet are included. Mandibular overjet can be interpreted and measured as a negative horizontal overjet (frequently seen in OI) as well. The same accounts for vertical overbite and anterior open bite: the latter can be measured as a negative vertical overbite.

The validated OHIP-49 questionnaire on oral health related quality of life was included in our standard set, instead of the more compact OHIP-14. This choice was based on the suggestion of the patient experts in our project team, with argumentation that the questions in this more comprehensive version reflected more of the issues that people with OI encounter. Both instruments are validated on the oral health related quality of life. If a more efficient tool for a quick assessment is required, OHIP-14 may be sufficient.

The use of a validated questionnaire to evaluate treatment will provide essential information on the impact of specific interventions. However, this was not included in this standard set.

‘Pain’ was mentioned as a top-priority item, during the focus group sessions. Participants mentioned tooth and jaw pain, but also earache. Pain is complex and can have multiple causes and other factors may contribute to, amplify or interfere with pain sensation. Without further specification and assessment of the pain, it was difficult to address it. In dentistry most common origins of pain are odontogenic pain (dental pain, pain of the teeth or surrounding tissues) or pain related to temporomandibular dysfunction.

A limitation of this study is the lack of children focus groups. Focus groups can successfully include children. However, it also creates challenges with e.g. need of adaptations based on maturity and age of the included children. Moreover, ensuring a comfortable and safe environment for children to express their opinions despite the power imbalances between adults and participants is of outmost importance. These issues need special competence of the investigating team with care taken to required considerations. The team stresses the importance of future studies investigating variables of importance for children's oral health related quality of life. Children focus groups would then be of significant value.

The authors acknowledge that in this study not all continents and countries were represented. The project initiators reached out to any known expert who had published about the topic, any expert center worldwide and any expert known to the patient organizations. Many experts have very small populations and their interest was often not significant enough to join the project or despite interest there were competing priorities as OI was only one out of many disorders they supported. The authors recognize that it is most essential that dentists are aware of OI and the consequences for oral health. It should also be underlined that there is a need of continuing networking to expand the knowledge of available expert teams and to enhance the collaborations between countries in the oral health care in OI, but also in other rare diseases.

## Conclusion

In this Delphi consensus study, a standard set of outcome measures and measuring instruments was developed for identification of prioritized oral health-related problems in OI. It is recommended to be implemented by dental practitioners in order to standardize and equalize the dental care in children and adults with OI. The minimum set of outcome measures ensures feasibility of use and requires only limited time from the clinician.

The use of a standardized set of outcome measures and measuring instruments will also facilitate future research and collaboration.

This is a first important step in composing a standard for OI specific oral healthcare. Further research and collaboration are necessary to identify and specify the oro-dental symptoms and problems of people with OI in broader perspective and eventually compose treatment guidelines, in order to improve the quality of care and oral health-related quality of life in individuals with osteogenesis imperfecta.

### Supplementary Information

Supplementary Material 1

## Data Availability

All data generated or analysed during this study are included in this published article.
