# Supplementary material for: A standard set of outcome measures for the comprehensive assessment of oral health and occlusion in individuals with osteogenesis imperfecta
Source: Orphanet J Rare Dis. 2024 Aug 13;19:294. doi: 10.1186/s13023-024-03308-5 (PMC11320983; doi:10.1186/s13023-024-03308-5)
Supplement: Supplementary file 1 — Supplementary Material 1 [file 13023_2024_3308_MOESM1_ESM.docx]

**Supplementary data**

| **Name** | **Country** | **Specialty** | **Affiliation** | **Focus (children, adults, both)** |
| --- | --- | --- | --- | --- |
| A. Ahmad | United Kingdom | Ped. Dentistry | Whittington Health NHS Trust Dental Services, London, UK | Children |
| K. Andersson | Sweden | Ped. Dentistry | Mun-H-Center, National Orofacial Resource Centre for Rare Diseases and Clinic of Pedodontics, Public Dental Service, Region Västra Götaland, Göteborg, Sweden  Department of Pediatric Dentistry, Institute of Odontology, Sahlgrenska Academy, University of Gothenburg, Göteborg, Sweden  Department of Dental Medicine, Division of Orthodontics and Pediatric Dentistry, Karolinska Institutet and Center for Pediatric Oral Health Research, Stockholm, Sweden | Children |
| H. Arponen | Finland | Orthodontics, sleep apnea and craniocervical pathologies | Department of Oral and Maxillofacial Diseases, University of Helsinki, Finland  Helsinki University Hospital Head and Neck Center, Helsinki, Finland | Both |
| L. Blokland | The Netherlands | Prosthodontist  Orofacial Pain and Dysfunction | Vogellanden, Center of Rehabilitation Medicine & Special Care in Dentistry, Zwolle, The Netherlands | Both |
| S. Colijn | The Netherlands | Patient representative | Care4BrittleBones Foundation, Wassenaar, The Netherlands  Elkerliek Hospital, Helmond, The Netherlands | - |
| H. Gjørup | Denmark | Orthodontics | Center for Oral Health in Rare Diseases, Department of Dental and Maxillofacial Surgery, Aarhus University Hospital, Aarhus, Denmark | Both |
| R. John | United Kingdom | Ped. Dentistry | Department of Paediatric Dentistry, University of Bristol, Bristol, UK | Children |
| M. Li | China | Ped. dentistry | Department of Stomatology, The University of Hong Kong-Shenzhen Hospital, Shenzhen, China | Children |
| S. Parekh | United Kingdom | Ped. Dentistry | Department of Paediatric Dentistry, UCL Eastman Dental Institute, London, UK | Children |
| J.M. Retrouvey | United States | Orthodontics | Department of Molecular Genetics, Baylor School of Medicine, Houston, TX, USA. | Both |
| T. Stutz Steiger | Switzerland | Patient representative | ProRaris, Switzerland | - |
| L. Zhou | China | Oral surgeon / implantology | Department of Stomatology, The University of Hong Kong-Shenzhen Hospital, Shenzhen, China | Both |
